# Magnetic Resonance Imaging Conditional Pacemakers: Rationale, Development and Future Directions

**DOI:** 10.1016/s0972-6292(16)30543-5

**Published:** 2012-09-01

**Authors:** Edmond M Cronin, Bruce L Wilkoff

**Affiliations:** Cleveland Clinic, Cleveland, Ohio

**Keywords:** magnetic resonance imaging (MRI), cardiovascular implantable electronic device (CIED), pacemaker, implantable cardioverter-defibrillator (ICD)

## Abstract

Pacemakers and other cardiac implantable electronic devices (CIEDs) have long been considered an absolute contraindication to magnetic resonance imaging (MRI), a crucial and growing imaging modality. In the last 20 years, protocols have been developed to allow MR scanning of CIED patients with a low complication rate. However, this practice has remained limited to a relatively small number of centers, and many pacemaker patients continue to be denied access to clinically indicated imaging. The introduction of MRI conditional pacemakers has provided a widely applicable and satisfactory solution to this problem. Here, the interactions of pacemakers with the MR environment, the results of MR scanning in patients with conventional CIEDs, the development and clinical experience with MRI conditional devices, and future directions are reviewed.

## Introduction

Magnetic resonance imaging (MRI) has emerged as a leading imaging modality, and continues to develop new and innovative uses. It offers peerless imaging of the heart, vasculature, brain, and other soft tissues, finding uses in almost every field of medicine, including electrophysiology. This is illustrated by the ever-growing number of scans: in 2007, 90.2 MRI scans per 1000 population were performed in the United States, up from 40.0 in 1997.[1] In parallel with the growth of MRI, the number of pacemakers and other cardiovascular implantable electronic devices (CIED) has also steadily increased. Each year, an estimated 200,000 patients in the United States receive a pacemaker; worldwide there are over 5 million pacemaker patients. An oft-quoted statistic is that an estimated 50-75% of CIED patients will require an MRI over the lifetime of their device.[2] However, given the potential for interaction between pacemakers and other CIEDs and the MRI environment, the presence of a pacemaker has long been considered an absolute contraindication to undergoing MRI.

## MRI-CIED interactions

Potential MRI-CIED interactions have been tested both in vitro and in vitro, and extensively reviewed. Briefly, these include:

1. *Translational attraction*: the static magnetic field can exert varying degrees of magnetic force and torque on the generator, with older models and ICDs being more susceptible at static field strengths up to 3 Tesla (T).[3-6] However, this is not likely of clinical significance, and we are not aware of any reports of this effect in humans.

2. *Heating*: this is of most concern at the lead tip, due to resistive heating at the lead-myocardium interface induced by both the radiofrequency (RF) current and gradient magnetic field. Temperature rises have been measured both in vitro and in an animal model, however other experiments have been more reassuring at scan settings in clinical use.[4,5,7,8] Significant heating at the lead tip would be expected to be accompanied by cardiac troponin isoform release and an increase in pace capture threshold, however this combination has been reported in only 1 of a total of 251 MRI scans in which cardiac biomarkers and threshold were prospectively measured.[4,10-12] Nonetheless, >1V increases in threshold have been reported in some series,[9,10] transient loss of capture in an animal model[5], and loss of capture with high impedance, troponin elevation, and delayed threshold increase have all been reported in CIED patients undergoing MRI.[13,14]

3. *Electrical current induction*: this has been demonstrated in vitro and in animal studies, due to both the RF field and pulsed gradients.[8,15] Rapid capture of the myocardium could result in hemodynamic compromise or ventricular fibrillation. While it has not been observed in humans, it could be the basis for some of the fatalities which have occurred in unmonitored pacemaker patients undergoing MRI.

4. *Electromagnetic interference (EMI)*: this phenomenon can be induced by the MR environment and can lead to incorrect pacemaker diagnostics, rapid ventricular pacing, or pacing inhibition.[5,17] Sensed EMI can be misinterpreted as atrial or ventricular high rate episodes. It can also lead to inhibition of pacing, which if prolonged, could be lethal to a pacemaker-dependent patient. Tracking modes can lead to rapid ventricular pacing. In ICDs, sensed EMI, if interpreted as ventricular tachyarrhythmia can lead to inappropriate therapies.[14]

5. *Reed switch behavior*: the static magnetic field leads to unpredictable reed switch position.[7,18-23] This is dependent on the patient's position relative to the bore and pacemaker orientation. Closure of the pacemaker reed switch leads to asynchronous pacing at the device specific "magnet rate", which, while a necessary feature, is undesirable for a prolonged period.

6. *Electrical or power-on reset*: this safety feature can be activated by battery depletion or EMI, and results in reprogramming to default (usually synchronous) settings. Devices which have undergone electrical reset can be reprogrammed and the reset may be without clinical consequence. However, in a pacemaker-dependent patient, reset to a synchronous mode, along with inhibition due to sensed EMI, would lead to asystole. Sub-threshold stimulation at the default output is also a potential hazard. Electrical reset was reported in 8/51 (16%) cranial MRI scans at 3T, but 7/115 (6.1%) and 3/555 (0.7%) studies at 1.5T.[4,10,24]

6. *Battery depletion*: a transient decrease in voltage is seen in many CIEDs post MRI, returning to close to pre-MR values at follow-up, which seems to be a temporary effect of increased current drain during the scan.[4,7,10,24] This is not suspected to be of clinical significance.

## MRI of patients with conventional (MRI unsafe) pacemakers

Despite these risks, MRI examinations are sometimes of critical diagnostic value to patients with pacemakers and other CIEDs, and several centers in Europe and North America have developed protocols for performing MRI in such patients.[4,7,9,10,23-25] Indeed, published reports exist on over a thousand patients with pacemakers and ICDs who have undergone MRI with a low but not zero complication rate. These protocols have been developed at centers with clinical and experimental expertise in the field and it is important to stress that the results obtained may not be applicable where such experience does not exist. In particular, the presence of personnel with expertise in (and not merely familiarity with) CIED interrogation, programming and troubleshooting is a pre-requisite, as well as those trained in advanced cardiac life support (ACLS). Continuous, real-time monitoring during the scan by visual and voice contact, and with pulse oximetry as well as ECG telemetry is necessary, as the MR environment causes sufficient electro-magnetic interference to render standard ECG tracings uninterpretable during scanning.[10,26] Generally, pacemaker-dependent patients' devices are programmed to an asynchronous mode, and non-pacemaker dependent patients' devices are programmed to "monitor" mode (OSO), to avoid competition with the intrinsic rhythm. Restrictions on scanning parameters, such as the use of transmit/receive rather than receive-only coils[27] and limits on specific absorption rate, also apply although the latter has been challenged.[28] Patients with abandoned and/or fractured leads are also excluded from MRI, as such leads are more susceptible to lead tip heating.[29] Patients with devices implanted less than 6 weeks previously have been excluded in many published series. Although it may be assumed that the concern is for translational attraction of ferro-magnetic components of the leads, there are no components of the lead that would be attracted by static or variable magnetic fields. The exclusion in the EnRhythm MRI study was to assure a stable a pacing capture threshold so that any changes that might be seen (none detected) would be more clearly caused by the interaction with the MRI environment. Uneventful MRI within hours of pacemaker implantation has been performed.[30]

Yet even with this largely positive experience, some risk remains, and unusual manifestations of pacemaker-MRI interactions continue to be reported.[13,17,31] With the large numbers of CIED components both current and historical, and the number of relevant patient factors, the number of permutations is vast. Efforts to track the safety or otherwise of MRI in large numbers of patients, such as the ongoing MagnaSafe Registry,[32] while important, will not be able to demonstrate safety across all CIED components and combinations of components. Regulators emphasize this aspect, and the need for adequately powered clinical trials.[33,34] Indeed, in the US, the Centers for Medicare and Medicaid Services will not reimburse healthcare providers for MRI scans performed in patients with a conventional CIED, unless it is part of a clinical trial or registry. The ideal solution is the development of CIEDs that are MRI conditional.

## MRI conditional pacemakers

A number of steps can be taken to render CIEDs less susceptible to the MR environment, including:

1. Reduction in the ferromagnetic content of the generator

2. Use of a Hall switch, which behaves in a predictable manner, instead of a reed switch

3. Modification of the leads to reduce lead tip heating

4. Shielding of the circuitry to render it immune to EMI

5. Protection of the internal power supply

6. Use of a dedicated MRI programming pathway to choose the appropriate pacing and sensing mode for the patient

MRI protocols and procedures can also be chosen to reduce the chances of interaction:

1. Lower static gradient field strength

2. Maximizing the distance between the CIED and the scanner

3. Limiting the RF power

These innovations have led to the development of several MRI conditional pacing systems, the first being Medtronic's RevoMRI system.

The publication of the EnRhythm MRI SureScan Pacing System Study was an important step towards the goal of rendering MRI broadly available to pacemaker patients.[35] This represented the first clinical data on a pacing system designed to function in the MRI environment. The pacing system, consisting of the EnRhythm MRI pulse generator and CapSureFix 5086MRI leads (Medtronic Inc., Minneapolis, MN) was implanted in 464 patients scheduled to undergo implantation of a pacemaker between 2007 and 2009. Patients were then randomized to undergo a non-clinically indicated MRI at 9-12 weeks post implant or not to undergo an MRI scan. The primary safety endpoint was the complication-free rate of the MRI procedure, and the primary efficacy endpoint compared pace capture threshold and sensing between the MRI and control groups. A total of 211 patients underwent an MRI and were followed for at least one month after the scan (mean ± SD 11.2 ± 5.2 months). Restrictions on the MRI scan similar to those frequently used in protocols for scanning conventional CIEDs were used: the static magnetic field strength was limited to 1.5T, maximum specific absorption rate (SAR) of 2W/kg and a maximum gradient slew rate of 200T/m/s. The isocenter of the RF transmitter coil was restricted to above C1 vertebra or below T12, meaning that in the trial, head and lumbar sequences were performed, although this restriction still permits imaging of most of the body. A dedicated MRI mode was programmed on, involving the completion of ten system integrity checks before the scan. Pacemakers were programmed to either asynchronous or non-stimulating mode, with output at 5V at 0.5ms. No MRI related complications were observed, although 8 patients reported mild, self-limited symptoms that were either related or of unclear relationship to the MRI scan. The primary efficacy endpoint was also met as there were no differences in threshold or sensing parameters post MRI. While more patients died in the MRI group than the group that did not undergo MRI (9 versus 2), 3 died before the MRI scan, and the remaining 6 died of various causes not clearly related to the implant or the MRI scan.

Renamed the RevoMRI SureScan system, it received Food and Drug Administration (FDA) approval in February 2011. While the current version used in the US is based on the EnRhythm pacemaker, which lacks several advanced features such as automated capture determination, a second generation system (Advisa DR MRI SureScan) is available in Europe and has received investigational device exemption (IDE) from the Food and Drug Administration to undergo clinical trials in the US. Due to redesign of the inner conductors the 5086MRI lead is one French size larger in diameter than the currently marketed CapSureFix Novus 5076 lead but is constructed more similarly to a legacy lead Medtronic 5068, which also has 2 filars and the same external diameter. The extendable helix can require more turns to be fully deployed. Sometimes the helix can extend suddenly from built up torque, and in our experience fluoroscopic monitoring of helix extension is required to ensure proper fixation. This may be responsible for a very slightly increased incidence of lead dislodgement noted in post-marketing performance surveillance (Medtronic, personal communication, March 2012). However the lead otherwise appears to have comparable reliability to the CapSureFix Novus 5076.

The other major pacemaker manufacturers also have MRI conditional devices either in use or in development. None are at present available in the US. Biotronik launched its Evia ProMRI pacemaker series, Safio 6.6F, Siello and Solia 5.6F MRI conditional leads (Biotronik SE & Co. KG, Berlin, Germany) in Europe in June 2010. St. Jude Medical's (Sylmar, CA, USA) Accent MRI pacemaker and Tendril MRI 6.6Fr leads received the Conformité Européenne (CE) mark in April 2011 for 1.5T whole body MRI with SAR up to 4w/kg, and are currently undergoing regulatory assessment in the United States. The MRI Activator™ remote control device can be used to activate and deactivate an MRI mode, consisting of pre-programmed MRI settings, in the MRI suite. This is designed to improve workflow by eliminating the need for cardiology personnel to be present before and after the scan. Boston Scientific (Natick, Massachusetts, USA) and Sorin Group (Milan, Italy) are also in the process of developing MRI conditional systems. Biotronik also launched the first MRI conditional ICD - the Lumax 740 - in Europe in November 2011.[36] Published, peer-reviewed clinical data are not yet available on the above systems, and none are available in the US.

Diagnostic quality is substantially affected when the heart, and to a lesser extent the thorax, is imaged, however when the CIED is outside the field of view artifact is not seen with either MRI conditional or conventional CIEDs.[37-39]

## Labelling and terminology

With the advent of MRI conditional pacing systems, a revised terminology was introduced by a panel representing multiple professional bodies.[40,41] Associated symbols for labeling and easy visual identification are presented in Table 1. It is noteworthy however, that the terminology is still applied incorrectly, even in the medical literature. [42] MRI conditional conveys the meaning that the CIED can be present in the MR environment subject to specific conditions, including limits on static field strength, gradient slew rate, RF fields, and anatomical limits on the isocenter of the RF transmitter coil. MRI conditional CIEDs have not been clinically tested outside of these parameters.

## Patient selection and economic impact

With the availability of MRI conditional pacemakers, how should patient selection for MRI conditional versus conventional pacemakers be approached? While in some cases, such as patients who require recurring MRI scans for follow-up of medical conditions, the choice is easy, this represents a relatively small group and in most cases the decision on which device to implant is more complex. In the US, the RevoMRI generator and leads, depending on contracting can be about 10% more costly. Therefore the strategy of implanting MRI conditional leads with a conventional generator, in anticipation of performing a generator change-out should an MRI be required in the future is weak. Use is also contraindicated in patients with conventional abandoned leads, which, as discussed above, present a higher risk than attached leads. While older patients have a higher short-term likelihood to encounter an indication to undergo an MRI scan than younger patients, the lifetime likelihood may be higher in younger patients. With the availability of different pacing features such as capture management in the RevoMRI device in the US, other clinical reasons might supercede the desire for an MRI conditional device. Currently, no specific guidelines have been developed to address this issue and the choice should ideally be made between the informed patient and his or her physician.

## Conclusions and future view

Although MRI can be performed with a low complication rate in patients with conventional, MRI unsafe, pacemakers, this practice requires detailed knowledge and expertise, as well as specific resources, and will likely remain restricted to a small number of expert centers. MRI conditional pacemakers provide a comprehensive solution and, even with the scanning restrictions on current devices, allow imaging of almost the entire body (Table 2). Future CIEDs are likely to be intentionally MRI conditional, but given the vast number of existing CIED components, MRI unsafe CIEDs will continue to present a dilemma for many years. MRI conditional technology will also have to keep pace with increasingly complex MR technology, including higher static field strengths, and clinical evaluation and relabeling of existing MRI conditional pacemakers may be necessary to achieve this.

## Figures and Tables

**Table 1:**
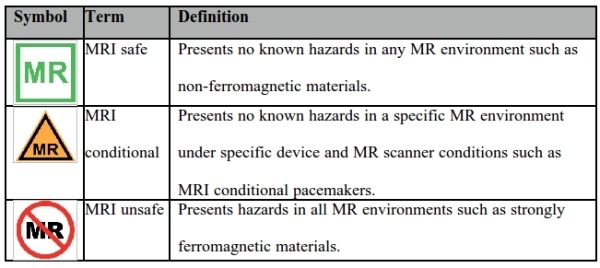
Current MRI terminology and labeling for pacemakers and ICDs. Adapted from reference 40

**Table 2:**
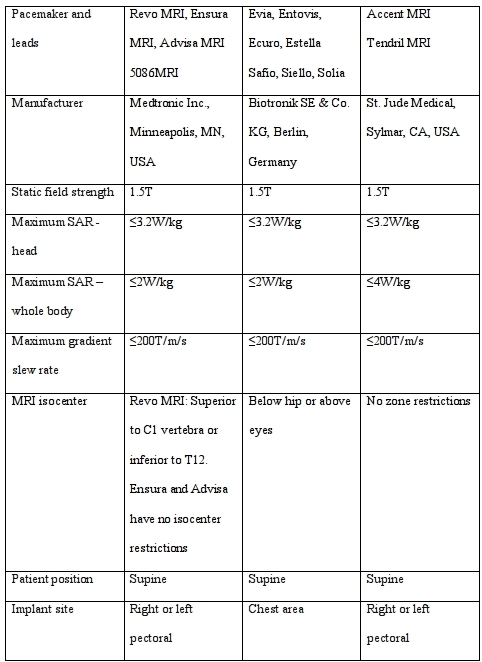
MRI conditions for current MRI conditional pacemakers. Only the Revo MRI system is available in the United States
